# The Impact of a 6-Week Nordic Walking Training Cycle and a 14-Hour Intermittent Fasting on Disease Activity Markers and Serum Levels of Wnt Pathway-Associated Proteins in Patients with Multiple Myeloma

**DOI:** 10.3390/jcm13102771

**Published:** 2024-05-08

**Authors:** Olga Czerwińska-Ledwig, Małgorzata Żychowska, Artur Jurczyszyn, Joanna Kryst, Jakub Deląg, Andżelika Borkowska, Joanna Reczkowicz, Tomasz Pałka, Przemysław Bujas, Anna Piotrowska

**Affiliations:** 1 Institute for Basic Sciences, Faculty of Physiotherapy, University of Physical Education, 31-571 Krakow, Poland; 2 Faculty of Health Sciences and Physical Culture, Biological Fundation of Physical Culture, Kazimierz Wielki University in Bydgoszcz, 85-064 Bydgoszcz, Poland; 3 Plasma Cell Dyscrasia Center, Department of Hematology, Faculty of Medicine, Jagiellonian University Medical College, 31-501 Krakow, Poland; 4 Doctoral School of Physical Culture Science, University of Physical Education, 31-571 Krakow, Poland; 5 Department of Bioenergetics and Physiology of Exercise, Medical University of Gdansk, 80-210 Gdansk, Poland; 6 Department of Physiology and Biochemistry, Faculty of Physical Education and Sport, University of Physical Education, 31-571 Krakow, Poland; 7 Institute of Sports, University of Physical Education, 31-571 Krakow, Poland

**Keywords:** multiple myeloma, Nordic walking, intermittent fasting, sclerostin, Dickkopf-related protein 1, osteoprotegrin, tartrate-resistant acid phosphatase 5b, Wnt pathway, vitamin D

## Abstract

**Background**: Multiple myeloma (MM) accounts for about 10–15% of all diagnosed hematologic malignancies and about 1–2% of all cancer cases. Approximately 80–90% of MM patients develop bone disease and the changes rarely regress. It is only possible to stop or slow their progression. A major role in bone destruction in MM is attributed to the Wnt signaling pathway, and its action can be modified by various types of interventions including training and diet. Therefore, the aim of this project was to evaluate the effects of a Nordic Walking (NW) training cycle and intermittent fasting (IF) on the levels of selected bone turnover markers associated with the Wnt pathway in patients with MM. **Materials and methods**: Results from 35 patients divided into training (NW and IF NW) and non-training (IF and control) groups were included in the analysis. A 6-week training cycle involving 60 min workouts 3 times a week was conducted. Body mass and composition as well as the levels of vitamin D, calcium and phosphorus, beta2-microglobulin, and albumin were examined before and after the completion of the training cycle. Markers of bone turnover were also determined: sclerostin (SOST), Dickkopf-related protein 1 (DKK-1), osteoprotegrin (OPG), osteopontin (OPN), and Tartrate-resistant acid phosphatase 5b (TRACP 5b). **Results**: There was no negative effect of IF or combined training and fasting on the nutritional status of the patients (the level of albumins was unchanged). Both training groups showed an increase in serum concentrations of the active metabolite of vitamin D (IF NW and NW: *p* = 0.001 and *p* = 0.022, respectively). The change in the concentration of this vitamin negatively correlated with the concentration of TRACP 5b (r = −0.413, *p* = 0.014). Evaluating the concentrations of markers related to bone turnover, a reduction in the concentrations of SOST (time: *p* = 0.026, time vs. group: *p* = 0.033) and TRACP 5b (time: *p* < 0.001, time vs. group *p* < 0.001) was indicated. **Conclusions**: The obtained results allow one to indicate the training with the poles as a safe and beneficial form of physical activity that should be recommended to patients suffering from MM. However, the results obtained in the present study are not sufficient to show the beneficial effect of IF applied without trainings.

## 1. Introduction

### 1.1. Multiple Myeloma

Multiple myeloma (MM) is a hematologic malignancy originating from plasma cells in the bone marrow. This disease constitutes approximately 10–15% of all diagnosed hematologic cancers and around 1–2% of all cancer cases [1]. MM primarily affects older individuals (median age at diagnosis is approximately 70 years) and is more common in men than women [2]. In patients, neoplastic transformation results in the formation of plasmocyte clones in the bone marrow producing, in typical cases, an antibody detected in serum and urine as a monoclonal protein. The presence of this protein and the activity of the cancer cells cause a number of organ-related consequences, upon which the diagnosis of MM is based. In the case of symptomatic MM, treatment is aimed at achieving disease remission [2].

In approximately 80–90% of MM patients, the development of bone disease occurs. This is a heterogeneous set of bone complications, most commonly involving pathological bone fractures, osteolytic lesions, as well as osteoporosis and osteopenia [2]. The onset of bone lesions is associated with cytokine and the receptor stimulation of bone cells by neoplastically altered plasmocytes. The interaction of malignant cells with bone tissue in the bone marrow microenvironment results in an increase in the activity of bone-depleting cells (osteoclasts) and a decrease in the activity of bone-forming cells (osteoblasts), which leads to the formation of the above-mentioned bone complications [3]. The serum concentrations of bone resorption-associated markers increase at the same time as concentrations of bone formation-related markers decrease [4]. Bone lesions in MM patients rarely regress; it is only possible to stop or slow their progression, both with treatment with bisphosphonate drugs, vitamin D, and calcium salt supplementation, as well as other preventive measures, including taking up physical activity [2].

### 1.2. Wnt Pathway in Multiple Myeloma

A significant role in bone destruction in MM has been attributed to the Wnt signaling pathway. Its activation by the classical pathway is associated with β-catenin, which is an effector protein of this pathway. This pathway has regulatory effects in many metabolic processes, and its dysfunctions have been observed in various types of diseases, including cancer [5]. This pathway plays an important role in the process of osteoblast differentiation [6]. Increased Wnt signaling can lead to elevated osteoprotegerin expression, resulting in a reduction in osteoclastogenesis and a slowing of bone resorption processes [7]. The regulation of the Wnt pathway is primarily based on inhibitors—acting directly on the receptor of this pathway (frizzled receptor and LRP 5 or 6 coreceptor) as antagonists, and proteins that bind Wnt proteins in the extracellular compartment [8]. The group of Wnt pathway inhibitors includes proteins such as Dickkopf-related protein 1 (DKK-1) and sclerostin (SOST), the other includes sFRP (soluble frizzled related protein) and WIF (Wnt inhibitory factor) proteins. In MM, this pathway is inactivated due to increased production of its inhibitors. This mechanism is thought to be responsible for the formation of osteolytic lesions in the bones of MM patients. This is confirmed by the fact that serum levels of DKK-1, SOST, sFRP2, and 3 correlate with the severity of bone lesions in patients [9].

The presence of the extracellular inhibitors of the Wnt pathway has been demonstrated in blood serum. One of them, DKK-1, is also secreted by multiple myeloma cells and plays a significant role in inhibiting the differentiation of osteoblasts during the course of the disease. It has been shown that the overexpression of DKK-1 and sFRP2 in MM cells is associated with the presence of osteolytic lesions in the bones observed on imaging techniques [10]. These proteins inhibit osteoblast differentiation [11,12]. SOST inhibits osteogenesis and enhances bone resorption. SOST levels are also positively correlated with serum phosphorus levels [13]. The modulation of SOST levels is the mechanism by which osteocytes coordinate osteogenesis in response to increased mechanical stimulation. This is likely through the release of local inhibition of Wnt signaling [14].

Proper Wnt signaling stimulates the expression of osteoprotegerin (OPG), and downregulates the expression of RANKL (Receptor Activator for Nuclear Factor κ B Ligand) in osteoblasts. RANKL binds to its receptor RANK on osteoclasts and precursor cells, leading to their differentiation and activation [15]. Osteoprotegrin, on the other hand, binds to RANKL and causes the inhibition of RANK receptor activation and, consequently, the NF-κB signaling pathway. The serum levels of RANKL and decreased levels of OPG have been shown in MM patients, indicating increased osteoclastogenesis and thus bone resorption [3,16].

### 1.3. Physical Activity and Wnt Pathway

Exercise can influence the regulation of bone formation processes by affecting the Wnt pathway [17]. In bone, as a result of shear stress on bone tubular fluid during exercise, integrin synthesis is stimulated, which induces the Wnt pathway in osteocytes and osteoblasts [4,17]. SOST expression in osteocytes and its serum concentration are regulated primarily by the mechanical load exerted on bone during exercise. It has also been shown that sclerostin expression is decreased when the bone is subjected to mechanical loading [18]. Mechanical stress leads to a decrease in the expression of this protein [6,14].

### 1.4. Intermittent Fasting

Intermittent fasting (IF), which involves maintaining the set time intervals of food intake, is being used increasingly for health purposes [19]. Under the influence of applied fasting, the body derives energy not only from liver glycogen, but also from ketones derived from adipose tissue [20]. It has also been shown that compared to a ketogenic diet, IF does not contribute to a decrease in bone mass [21]. Xu et al. indicated in their study in an animal model that a ketogenic diet alternated with IF every other day could lead to an improved balance between bone formation and bone resorption [22].

### 1.5. Nordic Walking

Marching with poles (NW, Nordic walking) is one form of physical activity recommended for both the elderly and patients with hematologic malignancies [23]. Previous studies have indicated the safety of NW training carried out in patients with MM [24]. However, there is a lack of studies evaluating the effect of this form of activity in MM patients on serum levels of biochemical markers related to the Wnt pathway.

### 1.6. Aim of the Study

We found no reports in the literature on changes in blood parameters related to Wnt pathway activity in MM patients associated with the use of IF protocols. The well-documented effect of physical activity on their changes prompted the authors of the project to take up the present research topic. Therefore, the aim of the study was to evaluate the effects of a 6-week cycle of walking-stick (NW) training and 14/24 intermittent fasting on disease-related blood parameters and body composition of patients with plasmocytic myeloma.

## 2. Material and Methods

### 2.1. Study Group Characteristics

**Figure 1:**
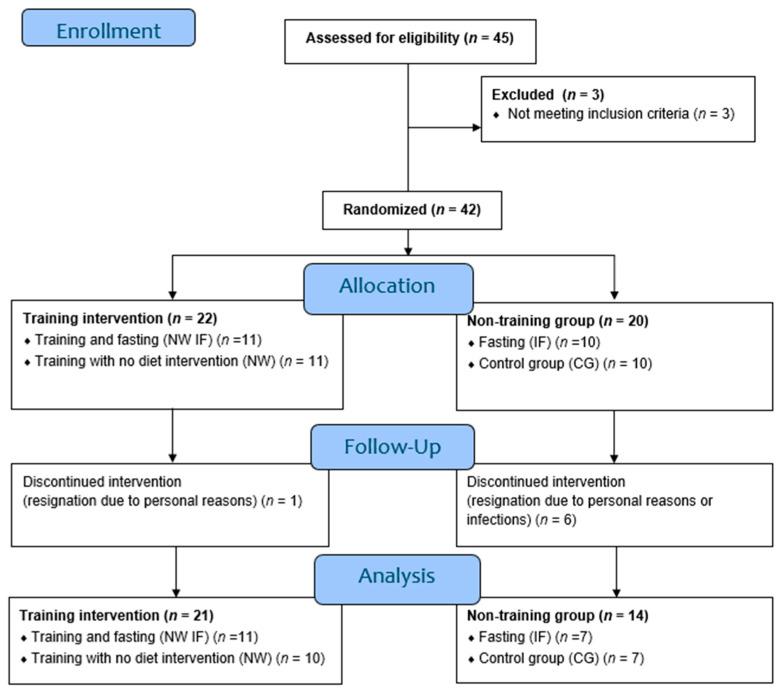
Consort patient flow diagram.

The study group selected from the group recruited to participate in the project consisted of 35 MM patients recruited among patients of the Department of Hematology, UJ CM, Krakow, Poland. The patients were randomly assigned (randomization) to 1 of 2 groups: those participating in Nordic walking training (training, finally included in the analysis: *n* = 21, M = 7, F = 14), and those not participating in training (non-training, finally included in the analysis: *n* = 14, M = 8, F = 6). Then, the two groups were divided into a fasting group (14/24 h intermittent fasting) and a non-fasting group. The characteristics of the study group are presented in Table 1 and the patient flow diagram is shown in Figure 1.

Inclusion was related to a thorough medical history. Inclusion criteria were plateau stage plasmocytic myeloma and the absence of contraindications to physical activity and undertaking intermittent fasting. All patients had undergone a bone marrow transplant procedure at least six months before participation. Each patient received zoledronic acid according to the guidelines of the International Myeloma Working Group [17]—4 mg by intravenous infusion every 4 weeks, as well as supplementation with 2000 U of vitamin D and 1000–1500 mg of calcium carbonate. Exclusion criteria were acute infections occurring during the course of the project, another malignancy, and falls from one’s own body complicated by trauma.

The subjects were informed in detail about the study protocol and the possibility of withdrawing consent to participate without consequences at any stage of the project. Approval from the bioethics committee (60/KBL/OIL/2022) was also obtained to undertake the study.

### 2.2. Study Protocol

Patients in the training group (n = 21) were subjected to a cycle of Nordic walking exercise classes held 3 times a week for a period of 6 weeks. A single workout lasted 60 min. In this group, 11 patients also undertook 14 h intermittent fasting with an 8 h eating window in which they took meals (IF NW), while the rest of the group did not change their eating habits (NW). In the non-training group (n = 14), half of the subjects undertook intermittent fasting (n = 7), and the remainder did not change their eating habits (n = 7). Measurements were taken in all subjects before participation in the study (I) and after 6 weeks of participation (II).

### 2.3. Methods

#### 2.3.1. Body Composition Analysis and Anthropometric Measurements

The following measurements were taken in all subjects before and after participation: waist circumference (WC) and hip circumference (HC) (using an anthropometric tape to the nearest 5 mm), and the results were used to calculate waist–hip ratio (WHR) and body height (BH) once. Body mass (BM) was also measured (using a Tanita BC 418 MA, measurement error: 0.1 kg, Tanita, Tokyo, Japan) and body composition was estimated using the bioimpedance method. The following were assessed for each person: body mass index—BMI (kg/m^2^); lean body mass—LBM (kg); soft tissue mass—SLM (kg); total body water—TBW (%); and percent body fat—FAT (%).

#### 2.3.2. Venous Blood Collections

Venous blood was collected from subjects by a laboratory diagnostician using a vacuum system (Vacutainer, BD) each time into two tubes with a clotting activator. To prevent fasting, on the day of the study, subjects were asked to eat a standardized, low-fat breakfast after a standard 8 h of fasting (non-fasting group) or 14 h of fasting (fasting group) before the blood draw.

#### 2.3.3. Measurements of Serum Concentrations of Biochemical Parameters

The determination of disease-related serum biochemical parameters (concentrations of calcium, phosphorus, vitamin 25(OH)D, beta-2-microglobulin, and albumin) were performed at an external medical diagnostic laboratory using common analytical methods.

Assays for concentrations of proteins associated with the Wnt pathway (SOST, DKK-1, OPG, OPN, and TRACP-5b) were performed by the mutliplex method using reagents from Biotechne (Minneapolis, MN, USA) and a Magpix Luminex Xmap instrument (Luminex Corporation, Austin, TX, USA). Samples were centrifuged after collection, and the serum was frozen at −80 °C until analysis.

#### 2.3.4. Nordic Walking Trainings

The training sessions were conducted outdoors by a qualified Nordic walking instructor in the fall and winter season. The intensity of the training was set to 60–70% of the HRmax calculated for each subject from the Nes formula [25], recognized in the literature as reliable for the older population. After consultation with the attending physician, the classical exercise test to determine the maximum heart rate was not performed due to the age of the patients and the nature of their disease.

Training intensity was monitored using sports-testers (M400 Polar, Kempele, Finland) with individually entered user data, including acceptable heart rate levels; if these were exceeded, the device beeped and the instructor corrected the subject’s exercise intensity.

### 2.4. Statistical Analysis

Descriptive statistics (mean, standard deviation or median, minimum and maximum value, respectively) were counted for all variables. Parameters characterizing the study groups were compared using the Chi2 test. Analysis of variance repeated measures ANOVA (RMANOVA) was then performed. For each indicator, the fulfillment of the test assumptions (sphericity by Maulchly’s test and homogeneity) was checked—if the sphericity condition was not met, the Greenhouse–Geisser (ε < 0.75) or Huynh–Feldt (ε > 0.75) correction was applied. The RMANOVA assessed the effects of time (measurement point), Group (group assignment) and the interaction of the two factors (Time*Group). The strength of the effect (eta^2^) was also assessed. For statistically significant results, post hoc test analysis was performed to refine the nature of the differences found (Bonferroni correction).

Correlations between studied parameters were also calculated. Parametric (Pearson’s r) or non-parametric (Spearman’s rho) coefficients were used depending on the results of the Shapiro–Wilk normality test.

The results *p* < 0.05 were considered statistically significant. Statistical analyses were performed using JASP 0.18.1 software (University of Amsterdam, Amsterdam, The Netherlands).

## 3. Results

### 3.1. Body Composition

In terms of the body composition parameters assessed and the results of somatic measurements, there were no statistically significant differences between groups and between measurement points. The results are shown in Table 2.

### 3.2. Blood Biochemical Parameters Related with MM

**Figure 2:**
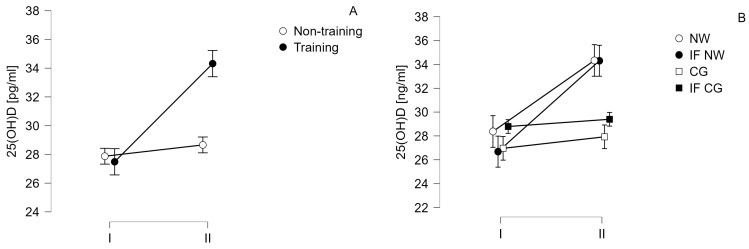
Change in 25(OH)D concentration over time (I—baseline; II—after 6 weeks) (**A**)—in training and non-training groups; (**B**)—in 4 study groups: CG—control group; IF CG—control group performing intermittent fasting; NW—group taking part in Nordic walking training cycle; IF NW—group taking part in Nordic walking training cycle and performing intermittent fasting.

No changes in calcium and phosphorus concentrations were observed between measurement points I and II in all study groups. For B2M, a slight decrease in concentrations was observed in all study groups, but nevertheless, these changes were not statistically significant (F = 0.920, *p* = 0.345, eta^2^ = 0.003, small effect size). Likewise for albumin concentrations, which increased slightly in both training groups, but without statistical significance (F = 2.447, *p* = 0.127, eta^2^ = 0.016, large effect size). Vitamin 25(OH)D concentrations increased significantly in the training-treated group (Figure 2). A post hoc test showed that differences emerged between the levels of the tested vitamin in subjects in the IF NW group before and after the training cycle (*p* < 0.001, t = −4.983). It also indicated that similar differences occurred in the NW group (*p* = 0.022, t = −3.715). Plasma vitamin D concentrations did not change significantly in the other groups. The described results for 25(OH)D as well as for other disease-related biochemical markers are summarized in Table 3.

### 3.3. Blood Biochemical Parameters Related with the Wnt Pathway

**Figure 3:**
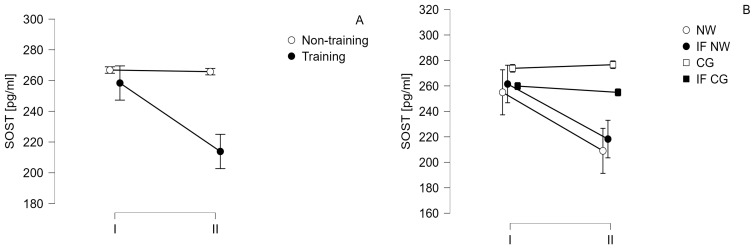
Change in sclerostin (SOST) concentration over time (I—baseline; II—after 6 weeks) (**A**)—in training and non-training groups, (**B**)—in 4 study groups: CG—control group, IF CG—control group performing intermittent fasting, NW—group taking part in Nordic walking training cycle, IF NW—group taking part in Nordic walking training cycle and performing intermittent fasting.

**Figure 4:**
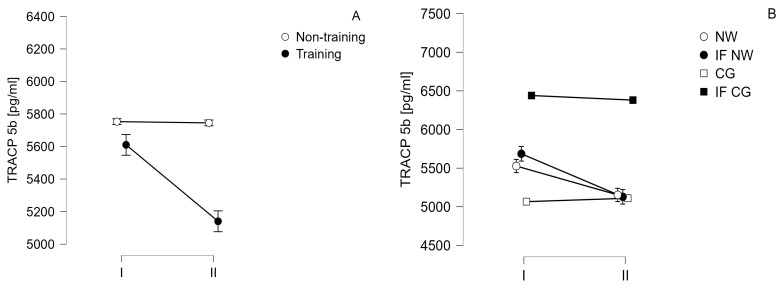
Change in tartrate-resistant acid phosphatase 5b (TRACP 5b) concentration over time (I—baseline; II—after 6 weeks) (**A**)—in training and non-training groups; (**B**)—in 4 study groups: CG—control group; IF CG—control group performing intermittent fasting; NW—group taking part in Nordic walking training cycle; IF NW—group taking part in Nordic walking training cycle and performing intermittent fasting.

The results of measurements of serum concentrations of biochemical indicators related to the Wnt pathway and bone turnover are summarized in Table 4 as well as Figure 3 and Figure 4.

Comparing training and non-training groups statistically significant differences were observed only for SOST (time—*p* = 0.026, F = 5.443, eta^2^ = 0.013, large effect size, time vs. group *p* = 0.033, F = 5.951, eta^2^ = 0.012, large effect size) and TRACP 5b (time—*p* < 0.001, F = 17.737, eta^2^ = 0.009, medium effect size, time vs. group *p* < 0.001, F = 16.623, eta^2^ = 0.008, medium effect size). Post hoc analysis showed the statistically significant decrease in SOST (*p* = 0.006, t = 3.602) and TRACP 5b (*p* < 0.001, t = 6.553) levels in the training group. Comparison between IF NW and NW groups did not show statistically significant differences between those groups for SOST. For TRACP 5b a statistically significant decline in NW (*p* = 0.030, t = 3.579) and IF NW (*p* < 0.001, t = 5.620) groups was observed.

### 3.4. Correlation Analysis of Wnt Pathway Markers

**Figure 5:**
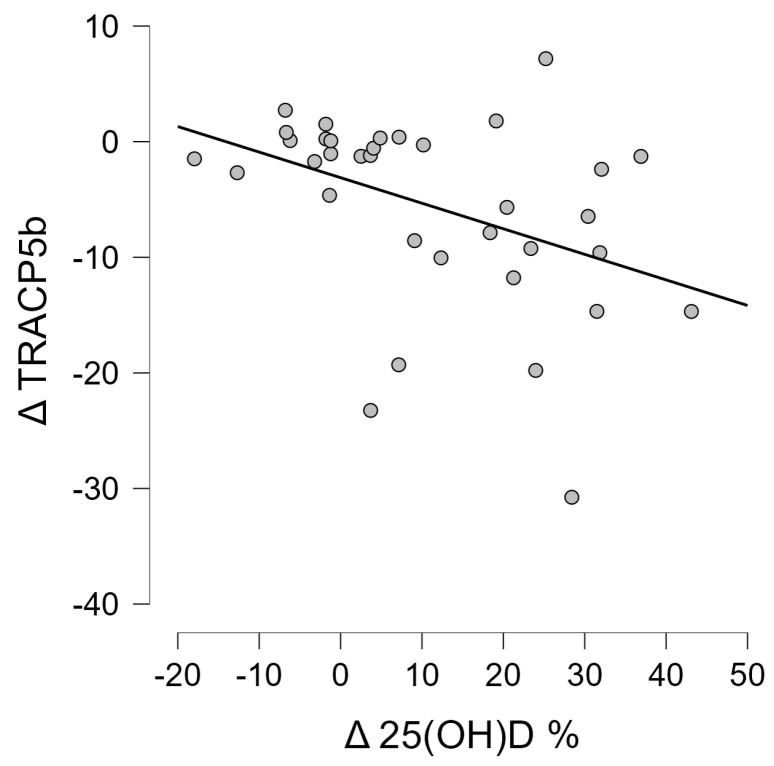
Correlation between the change in 25(OH)D levels induced after 6 weeks and the change in TRACP 5b protein levels observed after the same time in patients with multiple myeloma (r = −0.413, *p* = 0.014).

**Figure 6:**
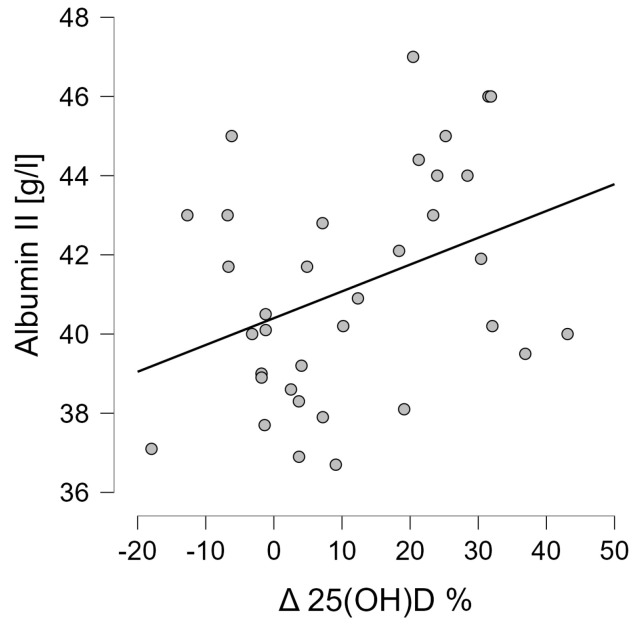
Correlation between the change in 25(OH)D levels induced after 6 weeks and the level of albumin captured at the end of the project (r = 0.367, *p* = 0.030).

**Figure 7:**
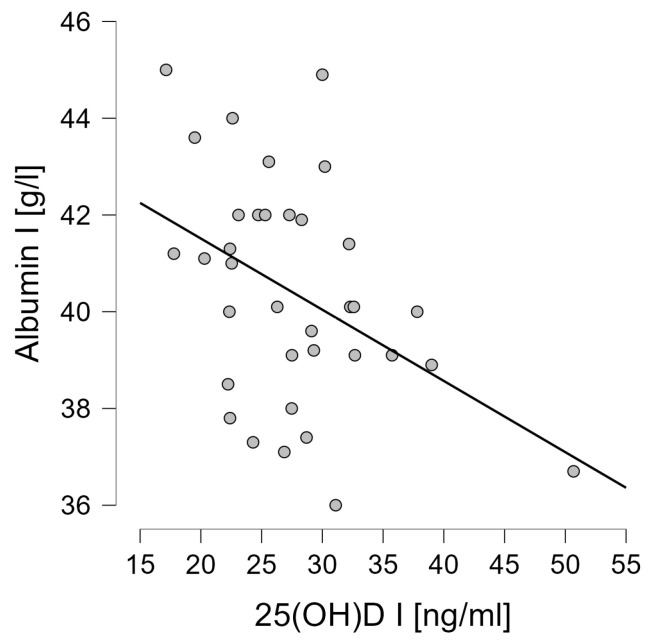
Correlation between the baseline level of 25(OH)D and the baseline level of albumin (r = −0.421, *p* = 0.012).

In the subsequent step, the correlations between the variables studied were tested. Baseline serum vitamin 25(OH)D levels did not correlate with any of the analyzed Wnt pathway markers. However, the presence of an inverse correlation was shown between the change in vitamin 25(OH)D concentrations that was induced by the proposed interventions and the change in TRACP 5b concentration (r = −0.413, *p* = 0.014) (Figure 5). It was also shown that the change in 25(OH)D concentration was positively correlated with albumin concentration after 6 weeks of intervention (r = 0.367, *p* = 0.030) (Figure 6) and baseline vitamin D concentrations correlated negatively with albumin concentration (r = −0.421, *p* = 0.012) (Figure 7).

No correlations between the studied biochemical markers related to the Wnt pathway and body weight and body composition were found.

## 4. Discussion

The present work, for the first time, shows how the combination of two interventions: dietary and exercise influences the levels of markers of Wnt pathway activity in patients with MM. Analyzing the data obtained, the main point to note is that intermittent fasting alone, nor intermittent fasting combined with exercise, did not contribute to changes in patients’ nutritional status or body composition. This observation is a favorable result and shows that the proposed interventions will not exacerbate involuntary changes in patients with MM. On contrary, the proposed interventions improved the concentrations of some bone formation-related markers analyzed in this study. Previous publications on exercise interventions in patients with MM or pre mm conditions also did not show significant differences in weight and body composition [26,27,28].

Among the evaluated parameters, vitamin 25(OH)D concentration is associated with disease but also directly related to bone metabolism. The effect of intervention in the form of 6-week NW training on 25(OH)D levels has been previously studied in patients with MM [24]. Our earlier work indicated that such intervention has a beneficial effect on the serum levels of this vitamin. Training sessions conducted in the spring–summer season resulted in a significant increase in mean 25(OH)D levels of 10.1 ng/mL. A significant decrease in calcium concentrations with no effect on serum phosphorus concentrations were also indicated. In the present study, when changes were observed after training conducted during the autumn–winter period (with much smaller insolation), an increase in 25(OH)D concentrations was also shown (in the whole training group by 6.8 ng/mL, and in the IF NW and NW groups by, respectively: 7.6 ng/mL and 6.0 ng/mL). However, no significant differences were shown in the IF group. Hence, we assume that the observed beneficial effect is solely due to the effect of the training stimulus applied.

Despite the fact that the training sessions were conducted during a period with significantly lower sunlight, the proposed outdoor physical activity induced favorable changes in 25(OH)D concentrations. This result is in contrast to the observations of Pilch et al. [29], where a statistically significant decrease in vitamin D concentrations was observed in women over 55 after 6 weeks of NW training conducted in the fall and winter. These differences are similar to those observed in the study by Pilch et al. [29], wherein significant decreases in body weight, BMI, and PBF were noted, a trend not observed in the current project. Another difference between the study of Pilch et al. and our present work is the vitamin D supplementation used routinely by MM patients securing the body’s needs, even with increased demand due to increased physical activity. On the other hand, properly administered supplementation, even with increased skin exposure to UV radiation during sunny days, will not adversely affect the body and does not risk exceeding safe serum concentrations [30,31].

An analysis of changes in the concentrations of bone turnover parameters indicated that significant differences were only observed for two markers, viz: TRACB 5b and SOST in the training group. After splitting the study group into the four groups (including dietary intervention), it was found that IF aided the decrease in TRACB 5b in the training group. The results for the IF NW group were slightly lower than those obtained for the NW group. The concentration of TRACB 5b in the IF NW group decreased by 10.7% and in the NW group by 5.5% (however, the differences between the changes were not statistically significant). The effect observed in the present project is important in relation to the use of dexamethasone in the combined MM pharmacotherapy models, and as reported in a study by Alrowaili et al. conducted in an animal model, this compound causes an increase in TRACP 5b levels [32], which may enhance osteolysis.

TRACP 5b, as described in the introduction, is a marker that indicates osteoclast activity, and its concentration correlates with the severity of MM bone disease. This protein is considered to be a good marker indicating the current activity of osteoclastic cells due to the fact that it is very rapidly degraded in the circulation [33]. This marker can be assessed by changing its concentration (as in the present project) or by evaluating the enzymatic activity of this protein.

As a result of anti-myeloma treatment, the concentration of TRACP 5b decreases significantly, as shown in the study by Terpos et al. [34]. This study also showed, as did the current project, no change in OPG levels after VAD (vikncristine, doxorubicin, and dexamethasone) + pamidronate therapy. The results of the current project show the same directions of change, which should be emphasized and pointed out as one of the strongest results of this observation.

Another marker of bone turnover that was significantly reduced in the training group was SOST. Dietary intervention had no effect on the concentration of this protein. The results of the IF group did not differ from the control group, while the results obtained for the IF NW group were not significantly different from the NW group. Such results suggest that the beneficial effect observed in the present project is exclusively related to the training intervention. SOST is one of the proteins that function as canonical inhibitors of the Wnt pathway. Together with the DKK protein, they are markers of osteocytes. The concentrations of these proteins are negatively correlated with osteoblastogenesis induced by the activity of the Wnt pathway, a beta-catenin-dependent canonical pathway [35]. Women undergoing 12-week physical training after breast cancer treatment showed statistically significant reductions in serum DKK-1 and sFRP-1 concentrations [36]. On the other hand, postmenopausal women with osteopenia subjected to a 12-week cycle of interval training on a cycloergometer taking place three times a week showed a reduction in serum SOST concentrations [37]. The results obtained in the present study are in agreement with previous observations on the effects of various training interventions on SOST serum concentrations. The only available publication-related MM where exercise intervention was applied and SOST serum levels were measured to date is the study by Seefried et al. [38]. In this study, 15 patients with pre-myeloma conditions monoclonal gammopathy of undetermined significance (MGUS) and smoldering multiple myeloma (SMM) were subjected to a cycle of 3 months of whole-body vibration training (WBVT) (with extension in 10 patients to 6 months). WBVT is a type of training considered to have a significant impact on the skeletal system and promote osteogenesis [39]. It is also the preferred type of training for people with contraindications to other forms of activity. The use of a vibratory stimulus that induces a tonic vibratory reflex amplifies the effect of the patient’s exercise on the vibrating platform [40]. A study by Seefried et al. [38] indicated that WBV training after both 3 and 6 months did not significantly affect the protein concentrations of SOST, TRACP 5b, DKK. Changes in serum calcium concentrations were also not observed. These results provide partial confirmation of the changes observed in the present project, where DKK and calcium also did not change significantly. However, the current study showed that the proposed exercise intervention reduced SOST and TRACT 5b concentrations. This indicates the superiority of stick training over vibration training in patients with MM or pre-myeloma conditions.

A correlation analysis indicated the presence of a positive correlation of the change in vitamin D concentration with albumin concentration. Albumin concentration can be regarded as a marker of the patient’s nutritional status. Therefore, it can be indicated that with the improvement of nutritional status, an increase in 25(OH)D concentration was observed.

Another correlation was between vitamin D and TRACP 5b. In this case, a significant negative correlation was indicated between the change in 25(OH)D concentration and the change in TRACP 5b concentration. This is an extremely favorable observation related to the pursuit of clinically beneficial changes involving increased vitamin D and the concomitant inhibition of TRACP 5b expression. Similar observations were made in a study by Miyakoshi et al. [41], where vitamin D was supplemented, and after a period of 6 months of supplementation, it was indicated that the serum concentrations of this vitamin increased and TRACP 5b decreased.

Despite the lack of significant results of IF in MM patients in this project, it is important to remember that IF may have a beneficial effect on cancer risk. In a study involving 14 individuals with metabolic syndrome, it was shown that 4-week IF (14 h a day from dusk to dawn) increased the serum concentrations of protein products of certain genes considered tumor suppressors and with DNA damage repair, as well as decreased concentrations of proteins that are promoters of carcinogenesis [42]. In animal models, a lower incidence of lymphoma [43] and acute lymphoblastic leukemia [44] has been indicated when IF is applied to a standard diet, but no effect on the incidence of acute myeloid leukemia has been observed [44]. IF used during anticancer therapy may contribute to its better tolerability and reduce the hematologic toxicity of drugs, as has been shown in breast cancer patients [45]. A study by Dorff et al. [46] indicates that, in women treated for gynecologic cancers, IF is safe and may contribute to reducing treatment complications (neutorpenia and polyneuropathy). The safety of IF in patients with various cancers, including hematologic cancers, is also indicated by Badar et al. [47]. A study by Marinac et al. [48], on the other hand, indicated that women after breast cancer who fasted for less than 13 h/day had a 36% higher risk of recurrence than those who fasted for more than 13 h. Based on our results presented above, the IF intervention was relatively short and the use of an extended period of intermittent fasting in the future could have a better effect, especially in combination with exercise. Given the favorable trends shown at this point, it would be beneficial to extend the fasting time in future studies involving MM patients.

## 5. Conclusions

The results of this study are of applicative importance. As the life expectancy of patients continues to increase due to new treatments, the introduction of physical activity and dietary interventions in patients with MM is important. It helps improve the quality of life and to maintain physical capacity. The use of the proposed exercise program alone or combined with intermittent fasting appeared to be safe for patients and had a positive effect a number of investigated blood parameters.

Considering the results obtained, no negative effect of intermittent fasting or combined training and fasting was shown on the patients in terms of the evaluated parameters. Among the routine parameters studied in the patients, an increase in the serum concentration of the active vitamin D metabolite was shown in both training groups. The other groups showed no significant changes. This allows us to conclude that the reason for the increase in vitamin D concentration was the training itself. Evaluating the concentrations of markers related to bone turnover, a favorable effect of training inducing a decrease in SOST and TRACP 5b concentrations was indicated. A negative correlation between 25(OH)D and TRACP 5b concentrations was also evident, another important argument showing the pleiotropic activity of this vitamin. Considering the positive trends observed, extending fasting duration in future MM patient studies could be more advantageous, especially combined with exercise.

## Figures and Tables

**Table 1:** Study group characteristics.

	Non-Training (n = 14)		Training (n = 21)	
	CG (*n* = 7)	IF (*n* = 7)	NW (*n* = 10)	IF NW (*n* = 11)
Age [years]	65.2 ± 4.8		65.4 ± 5.5	
	65.3 ± 5.0	65.1 ± 4.9	65.70 ± 6.9	65.18 ± 4.2
BMI [kg/m^2^]	28.6 ± 4.2		30.2 ± 3.5	
	29.0 ± 4.4	28.2 ± 4.4	28.6 ± 3.6	31.7 ± 2.8
Body height [cm]	166.1 ± 5.2		163.5 ± 10.3	
	164.9 ± 4.4	167.3 ± 5.9	164.6 ± 10.5	162.5 ± 10.6
MM duration [months]	56.8 ± 10.7		57.2 ± 12.8	
Disease duration [months]	54.4 ± 12.2	59.1 ± 9.2	57.5 ± 11.6	57.0 ± 14.3
Type of MM				
IgG:	(*n* = 8)		(*n* = 11)	
IgA:	(*n* = 5)		(*n* = 8)	
Other:	light chains (*n* = 1)		light chains (*n* = 2)	
Type of MM	κ-chains (*n* = 2)	κ-chains (*n* = 2)	κ-chains (*n* = 4)	κ-chains (*n* = 2)
	λ-chains (*n* = 1)	λ-chains (*n* = 3)	λ-chains (*n* = 2)	λ-chains (*n* = 3)
	κ-chains (*n* = 2)	κ-chains (*n* = 1)	κ-chains (*n* = 1)	κ-chains (*n* = 4)
	λ-chains (*n* = 1)	λ-chains (*n* = 1)	λ-chains (*n* = 1)	λ-chains (*n* = 2)
	light chains (*n* = 1)	-	light chains (*n* = 2)	-
Cycles of therapy	median = 6 (min = 3, max = 7)		median = 4 (min = 3, max = 8)	
Cycles of therapy	median = 6 (min = 3, max = 7)	median = 5 (min = 4, max = 7)	median = 4 (min = 3, max = 6)	median = 5 (min = 3, max = 8)
auto-HSCT	100% 75%—1 procedure 25%—2 procedures		100% 80%—1 procedure 20%—2 procedures	

Results are shown as mean ± standard deviation. CG—control group; IF—group performing intermittent fasting; NW—group taking part in Nordic walking training cycle; IF NW—group taking part in Nordic walking training cycle and performing intermittent fasting; BMI—body mass index, MM—multiple myeloma, Ig—immunoglobulins (type G, A) auto-HSCT—autologous hematopoietic stem cells transplantation. No statistically significant differences were found in study group characteristics.

**Table 2:** Body mass, body composition, and basic anthropometric characteristics of study groups.

	Time	Non-Training (*n* = 14)		Training (*n* = 21)	
		CG (*n* = 7)	IF CG (*n* = 7)	NW (*n* = 10)	IF NW (*n* = 11)
Body mass [kg]	I	79.4 ± 15.5		79.1 ± 12.3	
		79.1 ± 14.0	79.7 ± 18.1	80.6 ± 14.0	77.7 ± 11.0
	II	79.8 ± 15.6		79.0 ± 12.2	
		79.6 ± 13.6	80.1 ± 18.5	80.8 ± 13.9	77.4 ± 10.8
LBM [kg]	I	52.6 ± 9.8		51.4 ± 9.6	
		52.7 ± 10.1	52.5 ± 10.4	52.5 ± 9.1	50.4 ± 10.3
	II	55.2 ± 11.5		51.8 ± 9.6	
		56.5 ± 12.4	53.9 ± 11.4	53.3 ± 9.3	50.5 ± 10.2
SLM [kg]	I	50.0 ± 9.4		47.4 ± 9.0	
		50.1 ± 9.6	49.8 ± 9.9	48.5 ± 8.7	46.5 ± 9.7
	II	50.5 ± 9.7		47.9 ± 9.2	
		49.8 ± 9.1	51.1 ± 11.0	49.4 ± 9.1	46.5 ± 9.5
TBW [%]	I	46.4 ± 4.7		46.3 ± 4.6	
		46.3 ± 5.2	46.5 ± 4.4	46.4 ± 3.8	46.2 ± 5.5
	II	46.6 ± 4.6		47.0 ± 5.2	
		46.6 ± 5.5	46.6 ± 3.9	47.5 ± 5.5	46.5 ± 5.2
BMI [kg/m^2^]	I	28.6 ± 4.2		30.2 ± 3.5	
		29.0 ± 4.4	28.2 ± 4.4	28.6 ± 3.6	31.7 ± 2.8
	II	28.7 ± 4.3		30.2 ± 3.4	
		29.2 ± 4.4	28.3 ± 4.5	28.7 ± 3.6	31.4 ± 2.7
PBF [%]	I	33.1 ± 7.5		31.0 ± 3.9	
		32.9 ± 8.2	33.3 ± 7.3	30.9 ± 3.1	31.2 ± 4.7
	II	32.8 ± 6.2		30.9 ± 3.7	
		33.5 ± 8.0	32.2 ± 4.3	30.8 ± 2.9	31.0 ± 4.4
Waist circumference	I	97.6 ± 15.7		99.5 ± 10.9	
		98.3 ± 15.7	97.0 ± 16.9	101.8 ± 13.0	97.4 ± 8.8
	II	97.9 ± 16.1		97.6 ± 10.7	
		98.7 ± 15.8	97.1 ± 17.7	100.2 ± 13.6	95.3 ± 7.6
Hip circumference	I	103.7 ± 10.9		106.8 ± 8.9	
		106.2 ± 12.3	101.3 ± 9.5	106.9 ± 8.0	106.7 ± 10.1
	II	103.6 ± 10.8		105.2 ± 7.5	
		105.0 ± 12.2	102.2 ± 9.9	104.5 ± 7.1	105.8 ± 8.0
WHR	I	0.9 ± 0.1		0.9 ± 0.1	
		0.9 ± 0.1	0.9 ± 0.1	0.9 ± 0.1	0.9 ± 0.1
	II	0.9 ± 0.1		0.9 ± 0.1	
		0.9 ± 0.1	0.9 ± 0.1	0.9 ± 0.1	0.9 ± 0.1

Results are shown as mean ± standard deviation. CG—control group; IF—group performing intermittent fasting; NW—group taking part in Nordic walking training cycle; IF NW—group taking part in Nordic walking training cycle and performing intermittent fasting; LBM—lean body mass; SLM—soft lean mass; TBW—total body water; BMI—body mass index; PBF—percent body fat; WHR—waist–hip ratio. No statistically significant differences were found in body composition.

**Table 3:** Myeloma-related biochemical blood parameters.

	Time	Non-Training (n = 14)		Training (n = 21)	
		CG (n = 7)	IF CG (n = 7)	NW (n = 10)	IF NW (n = 11)
Ca [mmol/L]	I	2.37 ± 0.13		2.39 ± 0.15	
		2.36 ± 0.15	2.37 ± 0.13	2.39 ± 0.10	2.38 ± 0.18
	II	2.37 ± 0.10		2.38 ± 0.19	
		2.35 ± 0.11	2.41 ± 0.07	2.36 ± 0.12	2.39 ± 0.25
P [mmol/L]	I	0.97 ± 0.20		1.04 ± 0.24	
		0.95 ± 0.28	0.98 ± 0.12	1.01 ± 0.25	1.06 ± 0.22
	II	0.98 ± 0.19		1.04 ± 0.21	
		0.95 ± 0.24	1.03 ± 0.12	1.02 ± 0.20	1.07 ± 0.21
25(OH)D [ng/mL]	I	27.87 ± 6.11		**27.49 ± 7.10 ^#^**	
		26.96 ± 5.06	28.79 ± 7.31	28.37 ± 8.69 *	26.68 ± 5.61 *
	II	28.66 ± 7.31		**34.32 ± 10.07 ^#^**	
		27.92 ± 6.04	29.39 ± 8.83	34.33 ± 11.90 *	34.31 ± 8.67 *
B2M [mg/L]	I	2.15 ± 0.47		2.06 ± 0.36	
		2.20 ± 0.60	2.10 ± 0.35	2.12 ± 0.43	2.01 ± 0.30
	II	2.10 ± 0.44		2.02 ± 0.41	
		2.09 ± 0.54	2.12 ± 0.35	2.08 ± 0.45	1.97 ± 0.37
Albumin [g/L]	I	40.53 ± 2.06		40.30 ± 2.52	
		40.79 ± 1.81	40.27 ± 2.41	40.17 ± 2.67	40.41 ± 2.51
	II	40.75 ± 2.28		41.42 ± 3.20	
		40.99 ± 2.10	40.51 ± 2.59	41.58 ± 3.06	41.28 ± 3.46

Results are shown as the mean ± standard deviation. CG—control group; F CG—control group performing intermittent fasting; NW—group taking part in Nordic walking training cycle; IF NW—group taking part in Nordic walking training cycle and performing intermittent fasting; I—before, II—after 6 weeks; Ca—calcium; P—phosphorus; 25(OH)D—vitamin D active metabolite; B2M—beta-2 microglobulin; *, ^#^ *p* < 0.05.

**Table 4:** Wnt pathway-related proteins.

	Time	Non-Training (n = 14)		Training (n = 21)	
		CG (n = 7)	IF CG (n = 7)	NW (n = 10)	IF NW (n = 11)
SOST [pg/mL]	I	266.8 ± 100.2		258.4 ± 103.3	
		273.8 ± 93.0	259.8 ± 113.9	255.0 ± 106.2	261.5 ± 105.8
	II	265.5 ± 102.8		213.9 ± 86.0 *	
		276.1 ± 99.5	254.9 ± 112.8	209.0 ± 69.37	218.3 ± 102.1
DKK-1 [pg/mL]	I	4964.9 ± 906.0		4866.4 ± 1264.7	
		5252.2 ± 501.3	4677.5 ± 1155.2	4704.6 ± 1472.9	5013.5 ± 1093.9
	II	5099.4 ± 786.6		4845.5 ± 1362.2	
		5238.2 ± 591.9	4960.5 ± 972.3	4750.2 ± 1553.7	4932.0 ± 1233.4
OPG [pg/mL]	I	1155.9 ± 309.7		1096.6 ± 316.2	
		1039.4 ± 269.3	1272.5 ± 321.7	1109.1 ± 323.3	1085.3 ± 325.0
	II	1152.1 ± 312.7		1083.7 ± 262.4	
		1037.4 ± 266.2	1266.8 ± 332.1	1089.8 ± 248.3	1078.2 ± 286.6
OPN [pg/mL]	I	21,492.6 ± 9121.2		21,906.5 ± 7420.7	
		25,072.9 ± 5371.4	17,912.4 ± 9022.6	24,160.1 ± 6993.5	19,857.8 ± 7511.3
	II	21,328.3 ± 8940.6		19,849.1 ± 8418.0	
		25,313.1 ± 5038.6	17,343.4 ± 9523.8	21,344.2 ± 5906.7	184,890 ± 9298.4
TRACP-5b [pg/mL]	I	5752.8 ± 1375.1		5610.2 ± 1281.4	
		5065.9 ± 1398.6	6439.8 ± 1019.7	5527.2 ± 1333.6	5685.7 ± 1292.3
	II	5745.2 ± 1302.5		5140.3 ± 1169.5 *	
		5110.1 ± 1338.1	6380.4 ± 971.4	5153.2 ± 1182.5 *	5128.6 ± 1215.3 *

Results are shown as mean ± standard deviation. CG—control group, IF CG—control group performing intermittent fasting, NW—group taking part in Nordic walking training cycle, IF NW—group taking part in Nordic walking training cycle and performing intermittent fasting, SOST—sclerostin, DKK-1—Dickkopf-related protein 1, OPG—osteorotegrin, OPN—osteopontin, TRACP 5b Tartrate-resistant acid phosphatase 5b, I—before intervention, II—after intervention; * *p* < 0.05.

## Data Availability

Data are available upon request from the corresponding author.
